# The CT number accuracy of a novel commercial metal artifact reduction algorithm for large orthopedic implants

**DOI:** 10.1120/jacmp.v15i1.4597

**Published:** 2014-01-06

**Authors:** Guido Hilgers, Tonnis Nuver, André Minken

**Affiliations:** ^1^ Department of Medical Physics Radiotherapeutic Institute RISO Deventer The Netherlands

**Keywords:** metal artifact reduction, CT number accuracy, metallic hip prosthesis

## Abstract

Philips Healthcare released a novel metal artifact reduction algorithm for large orthopedic implants (O‐MAR). Little information was available about its CT number accuracy. Since CT numbers are used for tissue heterogeneity corrections in external beam radiotherapy treatment planning, we performed a phantom study to assess the CT number accuracy of O‐MAR. Two situations were simulated: a patient with a unilateral metallic hip prosthesis and a patient with bilateral metallic hip prostheses. We compared the CT numbers in the O‐MAR reconstructions of the simulations to those in the nonO‐MAR reconstruction and to those in a metal‐free baseline reconstruction. In both simulations, the CT number accuracy of the O‐MAR reconstruction was better than the CT number accuracy of the nonO‐MAR reconstruction. In the O‐MAR reconstruction of the unilateral simulation, all CT numbers were accurate within ±5HU (AAPM criterion). In the O‐MAR reconstruction of the bilateral simulation, CT numbers were found that differed more than ±5HU from the metal‐free baseline values. However, none of these differences were clinically relevant.

PACS numbers: 87.57.Q‐, 87.57.cp

## I. INTRODUCTION

In external beam radiotherapy treatment planning, CT numbers are used to perform tissue heterogeneity corrections,^1^, ^2^ When large metal objects are present in a CT study, which is the case for patients with pelvic malignancies and metallic hip prostheses, the CT numbers become corrupted by metal artifacts.

In 2012, Philips Healthcare (Cleveland, OH) released a novel metal artifact reduction algorithm for large orthopedic implants (O‐MAR).^(3)^ The commercial documentation clearly shows how O‐MAR improves the image quality by reducing the metal artifacts. However, little information is available about its CT number accuracy.

In this paper, we assess the CT number accuracy of O‐MAR using a phantom study. We simulated two situations: a patient with a unilateral metallic hip prosthesis and a patient with bilateral metallic hip prostheses. We compared the CT numbers in the O‐MAR reconstructions of these simulations to those in the nonO‐MAR reconstructions and to those in a metal‐free baseline reconstruction.

## II. MATERIALS AND METHODS

The phantom study comprised four scans, which were made with our Brilliance CT Big Bore (Philips Healthcare, Cleveland, OH). A cylindrical TomoPhantom (TomoTherapy Inc., Madison, WI) was used to simulate the pelvic area. This phantom has a diameter of 300 mm and is made of Solid Water (Gammex Inc., Middleton, WI; $p=1.04\;g/{\text{cm}}^{3})$. It contains 20 rods (d=28.5mm,l=70mm), which can be replaced with rods of other materials to simulate tissue heterogeneities.

**Figure 1:**
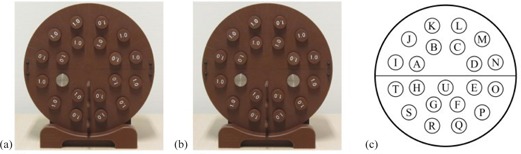
Set‐up for (a) the unilateral simulation scan, and (b) the bilateral simulation scan; locations of the volumes of interest (VOIs)(c) are shown in which the mean CT numbers and corresponding standard deviations were determined.

Prior to the first scan, the center of the phantom was aligned with the center of the bore. Then, a scout view was made to set the scan range. All scans used the same scan range to make sure that the scans and their reconstructions would geometrically coincide in order to facilitate data analysis. Subsequently, the four scans were made using our clinical scanning parameters (120 kVp, 250 mAs/slice, 2 mm slice width, HU range: −1024 to 3071, standard filter ‘C’ for filtered back projection). In the first scan, the phantom was scanned in its homogenous configuration. This scan was reconstructed without O‐MAR to obtain the metal‐free baseline reconstruction. For the second scan, we inserted a titanium rod ($\rho =4.51\;g/{\text{cm}}^{3}$) in the phantom (see Fig. 1(a)) to simulate a unilateral metallic hip prosthesis. In the third scan, the phantom contained an additional titanium rod (see Fig. 1(b)) to simulate bilateral metallic hip prostheses. From each of the simulation scans, two reconstructions were made: one without and one with O‐MAR applied. In the fourth and final scan, the phantom was scanned again in its homogenous configuration. This scan was reconstructed without O‐MAR to assess the reproducibility of the metal‐free baseline reconstruction.

In the metal‐free baseline reconstruction, cylindrical‐shaped volumes (d=20.0mm,l=42mm; approx. 6400 pixels) were delineated in each Solid Water rod (see Fig. 1(c)) with ProSoma v3.3 (MedCom GmbH, Darmstadt, Germany). Near the phantom center, one additional volume of interest (VOI) was created ('U'). Then, the VOIs were saved as a DICOM‐RT Structure Set, which was imported into the five other reconstructions. By doing so and because all scans geometrically coincided, we made sure that all VOIs geometrically coincided, as well. Subsequently, the mean CT numbers and corresponding standard deviations were determined in each of the VOIs for all reconstructions.

We used a *t*‐test to determine whether the mean CT numbers that were obtained from the reconstructions differed significantly from the mean CT numbers that were obtained from the metal‐free baseline reconstruction. Because of the large number of significance tests, a significance level (p) of 0.01 was chosen instead of 0.05 to prevent the identification of coincidental significance.

## III. RESULTS

The mean CT numbers and corresponding standard deviations from the reproducibility reconstruction were in excellent agreement with the baseline values; no differences larger than 0.6 HU were found and none of the differences were significant.

In the unilateral simulation, the mean CT numbers and corresponding standard deviations in the O‐MAR reconstruction were in closer agreement with the baseline values than those in the nonO‐MAR reconstruction (see Table 1). The range of mean CT numbers differences (Δμ) was −2.4 to 2.3 HU and −5.7 to 4.1 HU, respectively. As a result of the intrinsic nature of the t‐test and the smaller standard deviations in the O‐MAR reconstruction, more CT number differences were significant in this reconstruction (10/20) than in the nonO‐MAR reconstruction (8/20).

In the bilateral simulation, the mean CT numbers and corresponding standard deviations in the O‐MAR reconstruction were also in closer agreement with the baseline values than those in the nonO‐MAR reconstruction. The range of mean CT number differences (Δμ) was, respectively, −32.5 to 11.8 HU and −416.4 to 23.1 HU. The O‐MAR reconstruction contained less significant mean CT number differences (14/19) than the nonO‐MAR reconstruction (18/19).

**Table 1:** Results in Hounsfield units (HU)

	*Baseline*		*Unilateral Simulation*				*Bilateral Simulation*			
	*nonO‐MAR*		*nonO‐MAR*		*O‐MAR*		*nonO‐MAR*		*O‐MAR*	
*VOI*	μ(HU)	σ(HU)	Δμ(HU)	Δσ(HU)	Δμ(HU)	Δσ(HU)	Δμ(HU)	Δσ(HU)	Δμ(HU)	Δσ(HU)
A	26.6	18.8	−0.5	30.5	−0.3	13.9	**11.3**	39.4	**5.5**	18.2
B	23.0	19.0	2.3	**19.0**	**2.3**	7.7	**5.4**	34.3	**2.5**	14.7
C	32.4	19.3	0.6	17.5	−2.0	6.2	**5.1**	33.8	−1.9	13.9
D	33.8	18.9	1.0	14.3	0.6	5.3	**13.0**	38.5	**4.2**	17.8
E	32.0	18.8	−1.2	13.8	−2.4	5.1	N/A	N/A	N/A	N/A
F	25.3	19.4	**2.6**	18.6	−0.4	7.2	**23.1**	41.2	**11.5**	18.3
G	28.3	19.7	−3.3	28.2	−0.8	11.3	**20.9**	38.5	**11.8**	17.5
H	33.0	18.9	N/A	N/A	N/A	N/A	N/A	N/A	N/A	N/A
I	25.9	15.1	0.3	26.0	0.8	9.8	**10.4**	30.8	**6.0**	12.5
J	22.0	17.5	0.9	14.6	0.9	5.5	**1.7**	23.5	−0.1	9.5
K	28.6	15.4	0.4	16.3	−0.6	6.3	0.2	24.3	−0.5	10.2
L	30.7	15.6	0.3	15.0	**0.9**	5.5	**1.4**	24.5	0.2	10.2
M	33.2	15.5	−1.6	15.5	**1.2**	5.4	**2.1**	26.4	−2.0	11.5
N	27.4	15.0	0.3	12.6	0.2	4.2	**9.9**	30.5	**4.3**	12.9
O	31.9	15.3	−1.2	11.5	−1.9	4.0	−154.9	38.6	−32.5	16.9
P	20.5	16.0	**4.1**	11.5	**1.6**	4.3	**17.0**	36.0	**7.2**	15.7
Q	26.0	16.4	−0.1	15.9	**1.2**	5.7	**2.7**	27.1	0.6	11.4
R	25.5	16.0	0.2	17.4	−1.2	7.0	**3.0**	29.6	−0.7	12.5
S	28.6	15.6	0.9	30.6	0.3	13.0	**13.3**	34.9	**4.5**	15.7
T	24.7	15.3	−5.7	31.9	−1.4	13.6	−152.1	41.7	−31.5	18.3
U	18.7	26.9	−4.7	27.4	−1.0	10.1	−416.4	297.5	−23.7	25.5

For each volume of interest (VOI), the mean CT number (μ) and standard deviation (σ) in the metal‐free baseline are listed, as well as the differences with respect to these values (Δμ,Δσ) in the other reconstructions. Significant differences in mean CT numbers (p<0.01) are printed in **bold**. For the locations of the VOIs, see Fig. 1(c).

N/A=not applicable.

## IV. DISCUSSION

In our clinic, all patients who are to receive external beam radiotherapy, are imaged with 120 kVp and 250 mAs/slice — the kVp and mAs choices in this paper. Different kVp and mAs values could potentially yield other results. Effects of kVp and mAs changes on the effectiveness of O‐MAR are discussed by the manufacturer in a white paper.^(3)^

Although in the metal‐free baseline reconstruction all VOIs contained the same material (i.e., Solid Water), the mean CT numbers varied more than expected. Additional measurements pointed out that the composition of the rods was less consistent than thought beforehand.

In the two O‐MAR reconstructions, the mean CT numbers and corresponding standard deviations were in better agreement with the metal‐free baseline values than in the two nonO‐MAR reconstructions. Thus, the CT number accuracy of an O‐MAR reconstruction is better than the CT number accuracy of a nonO‐MAR reconstruction. In both O‐MAR reconstructions, the amount of significant mean CT number differences was considerable — 10/20 and 14/19 in the unilateral simulation and the bilateral simulation, respectively.

**Figure 2:**
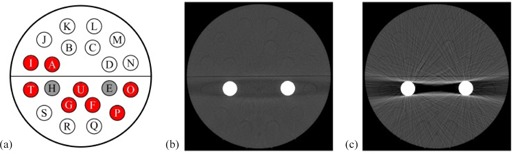
Results of the bilateral simulation: (a) the volumes of interest (VOIs) in the O‐MAR reconstruction of the bilateral simulation with a significant (p<0.01) mean CT number difference larger than 5 HU in red; (b) a slice in the O‐MAR reconstruction showing the residual artifact around which all of these differences are located; and (c) the same slice in the nonO‐MAR reconstruction showing the original artifact. Both CT images have the same window width (300) and level (1200).

For the CT number accuracy of water, AAPM Task Group 66^(2)^ has defined a tolerance of ±5HU. When we apply this tolerance to our results, the mean CT numbers in the O‐MAR reconstruction of the unilateral simulation are all accurate, as the largest significant mean CT number difference is 2.4 HU. In the O‐MAR reconstruction of the bilateral simulation, however, mean CT number differences larger than 5 HU can still be found (see Fig. 2(a)). All of these are situated in or near to the residual artifact between the two titanium rods (see Fig. 2(b)), which is a remainder of the characteristic artifact that is normally observed between two metallic inhomogeneities (see Fig. 2(c)).

The relatively large mean CT number differences in ‘O’ (−32.5HU) and ‘T’ (−31.5HU) can be considered clinically irrelevant, because it is good practice to choose beam arrangements in which the metallic prostheses are avoided.^4^ From the remaining VOIs, ‘U’ showed the largest mean CT number difference (−23.7HU). In a worst case scenario, in which a 5cm×5cm beam diagonally crosses the residual artifact, while avoiding the metallic prosthesis, the distance that this beam travels through the artifact is 60 mm. Assuming a difference of 25 HU along this distance, the radiological path length will be off by 1.5 mm. This is comparable to the CT pixel size and smaller than the dimensions of the dose grid and the uncertainties in structure delineation by the radiation oncologists. Therefore, we do not consider any of the significant mean CT number differences in the O‐MAR reconstruction of the bilateral simulation as clinically relevant. Our results are consistent with the findings of Li et al.,^(5)^ who evaluated the CT number accuracy of O‐MAR in clinical patient scans.

Several months after the initial experiment, additional measurements were performed. The bilateral simulation was repeated in its original form (titanium rods in ‘H’ and ‘E') and two alternative forms (titanium rods in ‘A’ and ‘E', and ‘R’ and ‘K'). Both the results of the repetition and the results of the alternative simulations were consistent with our earlier results.

Li et al.^(5)^ also performed a phantom study in which they obtained similar results and conclusions. They used a different phantom containing inserts with varying electron densities. Also, their CT scan settings and analysis methods were different. We designed a well‐controlled experiment in which we used a homogenous phantom and only introduced titanium rods. We finished by checking the reproducibility of the first scan. Therefore, we can attribute any HU value changes to the presence of the titanium rods and O‐MAR. Moreover, we have tested our results several months later for reproducibility and for multiple configurations of the titanium inserts.

## IV. CONCLUSIONS

In this paper, we assessed the CT number accuracy of O‐MAR. We simulated a patient with a unilateral metallic hip prosthesis, as well as a patient with bilateral metallic hip prostheses. In both simulations, the CT number accuracy of the O‐MAR reconstruction was clearly better than the CT number accuracy of the nonO‐MAR reconstruction.

Compared to a metal‐free baseline, the O‐MAR reconstruction of the unilateral simulation provided accurate CT numbers. In the O‐MAR reconstruction of the bilateral simulation, we found CT number differences that were larger than 5 HU due to a residual artifact. However, these differences are not of clinical relevance.
